# Dynamics Changes in Basal Area Increment, Carbon Isotopes Composition and Water Use Efficiency in Pine as Response to Water and Heat Stress in Silesia, Poland

**DOI:** 10.3390/plants11243569

**Published:** 2022-12-17

**Authors:** Barbara Sensuła, Sławomir Wilczyński

**Affiliations:** 1 Institute of Physics-Center for Science and Education, The Silesian University of Technology, Konarskiego 22B, 44-100 Gliwice, Poland; 2 Department of Forest Ecosystem Protection, the University of Agriculture in Krakow, Al. 29 Listopada 46, 31-425 Kraków, Poland

**Keywords:** pine, BAI, isotopes, iWUE, water and thermal stress, SPEI, Poland

## Abstract

Trees can be used as archives of changes in the environment. In this paper, we present the results of the analysis of the impact of water stress and increase in air temperature on BAI and carbon stable isotopic composition and water use efficiency of pine. Dendrochronological methods together with mass spectrometry techniques give a possibility to conduct a detailed investigation of pine growing in four industrial forests in Silesia (Poland). Detailed analysis-based bootstrap and moving correlation between climatic indices (temperature, precipitation, and Standardized Precipitation-Evapotranspiration Index) and tree parameters give the chance to check if the climatic signals recorded by trees can be hidden or modified over a longer period of time. Trees have been found to be very sensitive to weather conditions, but their sensitivity can be modified and masked by the effect of pollution. Scots pine trees at all sites systematically increased the basal area increment (BAI) and the intrinsic water use efficiency (iWUE) and decreased δ^13^C in the last century. Furthermore, their sensitivity to the climatic factor remained at a relatively high level. Industrial pollution caused a small reduction in the wood growth of pines and an increase in the heterogeneity of annual growth responses of trees. The main factors influencing the formation of wood in the pines were thermal conditions in the winter season and pluvial conditions in the previous autumn, and also in spring and summer in the year of tree ring formation. The impact of thermal and pluvial conditions in the year of tree ring formation has also been reflected in the isotopic composition of tree rings and water use efficiency. Three different scenarios of trees’ reaction link to the reduction of stomata conductance or changes in photosynthesis rate as the response to climate changes in the last 40 years have been proposed.

## 1. Introduction

In recent decades, it has been observed that a significant problem of increase in temperature and decrease in precipitation is responsible for the drought period in most ecosystems all around the world. Scientists have used many different indices and different archives have been examined to monitor the impact of water stress on forest stands [1,2,3,4,5,6,7,8]. Pines and their tree ring properties can be used as archives of the change in the environment in which the tree has been growing. The climatic changes and the anthropogenic effect can be recorded, e.g., in the width of the tree ring, and its isotopic and elemental composition [1,2,3,4,5,6,7,8,9,10,11,12,13]. 

It is well-known that the influence of seasonal fluctuation in temperature and water stress, which reduces the stomata conductance, changes the photosynthetic rates, and has an impact on tree-ring width, stable isotopic composition, and thus on water use efficiency. Several models of stable carbon isotope variation during photosynthesis (e.g., [3]) describe fractionation that occurs due to diffusion in air and stomatal conductance, and fractionation caused by carboxylation (discrimination by RuBisCO). The pathway and associated fractionation of carbon isotopes in CO_2_ during photosynthesis and respiration have been described in detail [2,3,6,12,13]. Through photosynthesis, plants convert CO_2_ and H_2_O to saccharides (C_6_H_12_O_6_)_n_, using light, and release oxygen into the atmosphere. Tree adaptation and its sensitivity to climate change can be studied using statistical methods, including bootstrap correlation and moving correlation functions [11,14]. The comparison of dynamic variation in tree ring width and stable carbon isotopic composition, and thus in the relationship between stomata conductance (g) and photosynthesis rate (A), and thus in water use efficiency iWUE (iWUE = A/g) of trees with variability of climatic factors examines how trees react and adapt to climate changes, including water stress and increased air temperature increase [9,15,16]. Previous analysis has shown that climatic signals can be modified or masked by human activities (for example, an increase in the emission of pollution) that have an impact on the ecosystem in which the tree is grown. The input of pollutants into the atmosphere has increased significantly since the middle of the twentieth century as a result of the increase in industrial activities in the world. Since the 1980s, pro-ecological policy and pro-ecological investments were implemented in most of the factories in Poland. The further reduction of the emission of contamination in Silesia was associated with restrictive governmental regulations on emissions according to EU legislation.

To date, despite interest in research on trees as bioindicators, few studies have considered the influence of climate on pine stands and stable isotopic composition of trees growing in forests localized in highly populated and industrialized parts of southern Poland- in Silesia, e.g., [7,8,14,17,18,19,20]. This study explores, for the first time, the interaction between tree properties and the index: Standardized Precipitation-Evapotranspiration Index (SPEI) [21,22,23,24]. The aim of this work is to broaden our understanding of spatial and temporal variations in BAI, carbon isotope fractionation, and iWUE associated with water balance and increased temperature. Therefore, we investigate the relation between seasonal fluctuation in the amount of precipitation and potential evapotranspiration and the response of trees to these changes in the selected region. 

Annual wood growth can be considered a measure of the sensitivity of trees to pressure from various environmental factors, including climatic conditions [25]. An important factor that influences the variation in stem growth in trees is changing weather conditions from year to year [26]. One of the parameters for the vitality of trees can be, for example, the increment of the basal area, which provides information about the amount of wood deposited by trees. Dendrochronological methods can be used, for example, to demonstrate the influence of individual climatic elements on the growth of wood, that is, the vitality of trees [27]. However, industrial pollution often reduces the vitality of trees, the growth of trees, and their sensitivity to meteorological factors, and increases the heterogeneity of their annual growth responses [28,29,30,31,32]. Most of the dendrochronological studies of tree rings evaluate changes in radial stem growth, which in coniferous species is characterized by a clear decrease with age, which may obscure the true causes of changes in tree condition. Unlike radial growth, basal area increment (BAI) in healthy trees is characterized by a long-term upward trend with age, reaching its peak at several dozen years of age [33]. In particular, we investigate whether BAI is a sensitive indicator of tree vigor.

The purpose of these studies was to investigate the sensitivity of the pine population to temperature and precipitation in four forest areas in Silesia influenced by industrial pollution. The annual variation of the basal area increment, δ^13^C, and iWUE were used as the indicators of the tree’s response to climate change, when there was a strong increase in industrial activities in the investigated area.

## 2. Results

**Figure 1:**
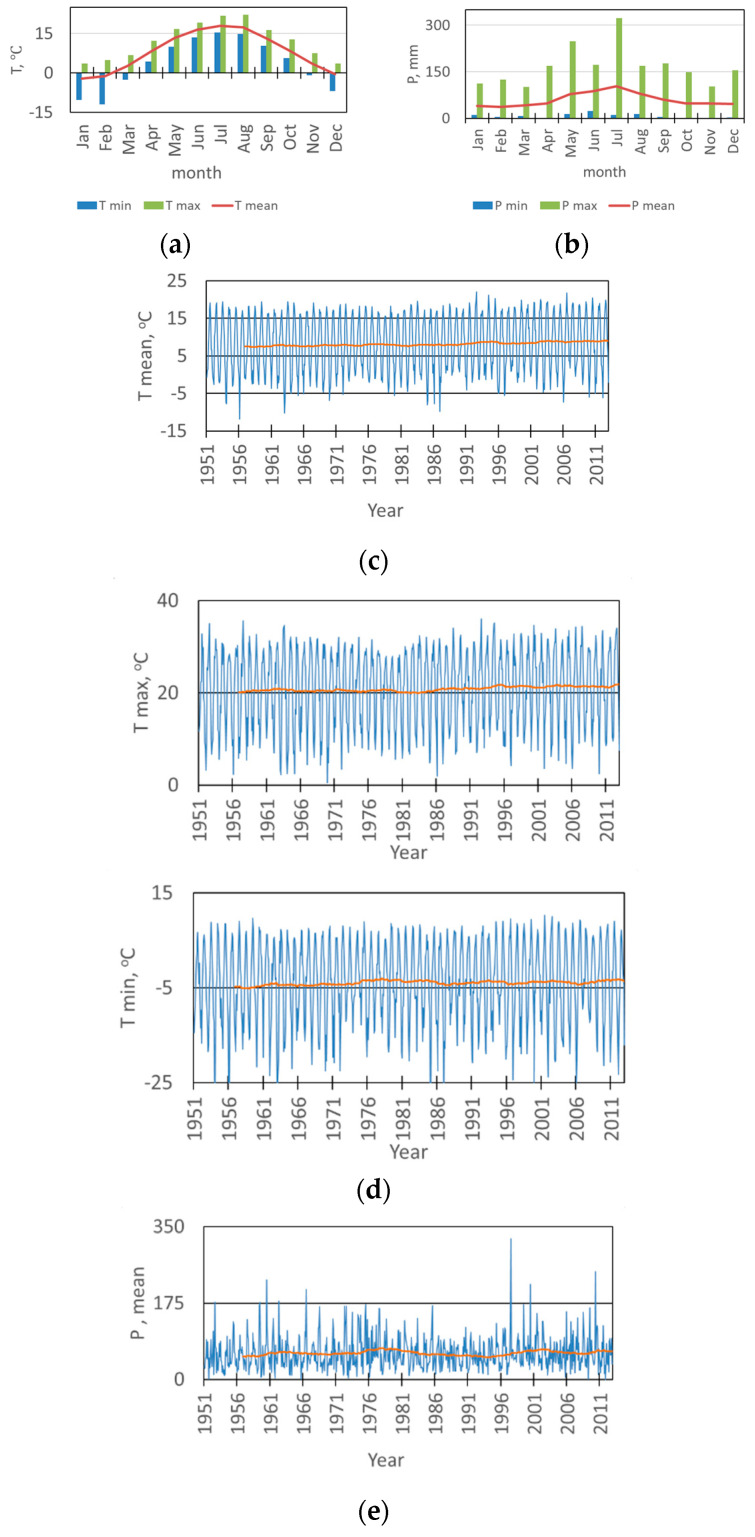
Trends and intraannual variation in air temperature (T) and amount of precipitation (P) mean value (mean) and extremes (max-maximum, min-minimum) (**a**,**b**) and comparison of time series, and trends in the total amount of precipitation and mean, maximum (max) and minimal (min) air temperature, respectively, orange lines indicate a trend of 5-year mean of average temperature (**c**), maximal and minimal temperature (**d**) and the sum of precipitation (**e**), in the investigated area (1950–2012).

Figure 1 shows dynamic changes in the average monthly temperature and the amount of precipitation in the investigated area. The period from 1951 to 2012 was characterized in the regional climate records by an annual mean temperature range of 6.5 to 10.5 °C) and a mean annual precipitation range of 360 to 870 mm. 

**Figure 2:**
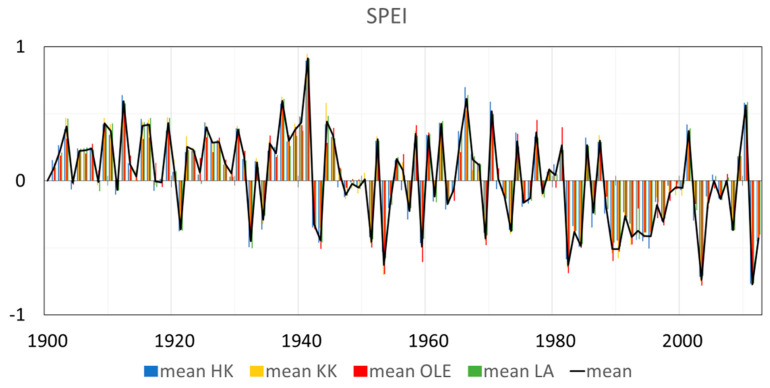
Comparison of time series and trends of annual mean SPEI between 4 investigated sites in Poland: HK-Dabrowa Górnicza, LA-Łaziska Górne, KK-Kędzierzyn Koźle, OLE-Olesno. Based on the data set [23,24].

**Figure 3:**
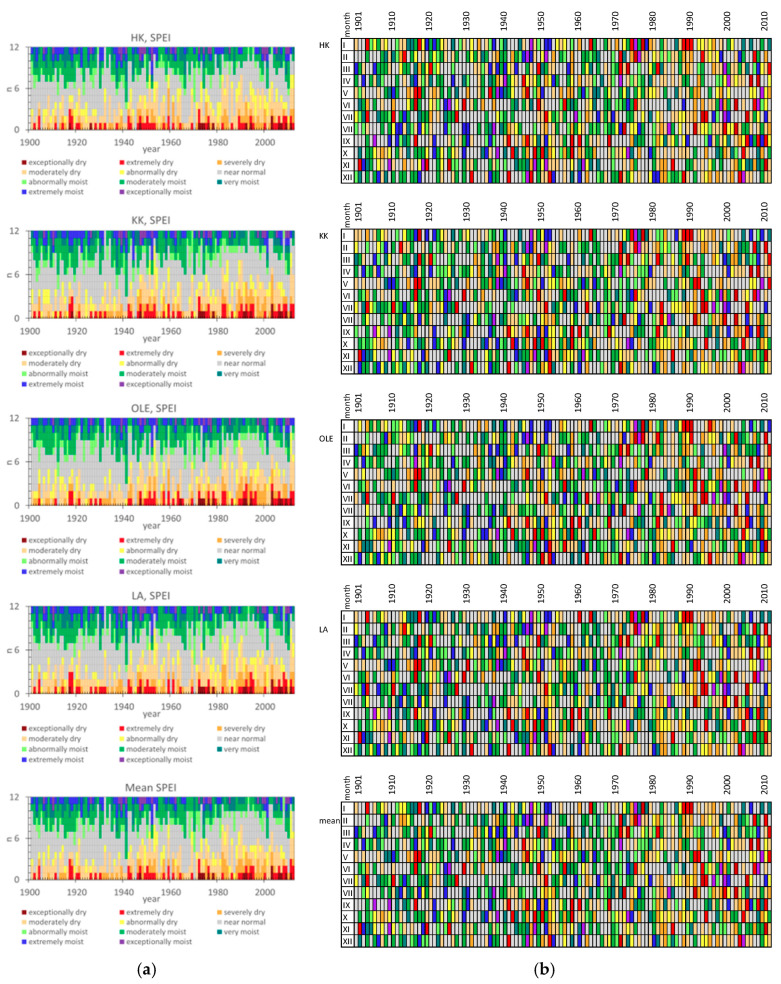
Graphical analysis of SPEI in the investigated area (HK-Dabrowa Górnicza, LA-Łaziska Górne, KK-Kędzierzyn Koźle, OLE-Olesno and mean SPEI value in Silesia region) since 1900–2012. Comparison of time series, means, extremes, and trends of monthly SPEI (**a**) the number of months in each year (1900–2012) with the division to 11 types of the period depends on SPEI (**b**) the temporal distribution of variation in SPEI over each year since 1900–2012. The comparison according to the threshold and symbology for the SPEI [24], and data [23].

Figure 2 and Figure 3 provide an overview of the data from the SPEI Global Drought Monitor related to the climatic water balance, including a comparison of time series, means, extremes, and trends. The threshold and symbology for the SPEI were used according to the Global Operational Support Team [24]. To detect drought episodes, it is possible to use SPEI, which takes into account the intensity and duration of drought episodes. The idea behind SPEI is to compare the maximum possible atmospheric evaporation demand with current water supplies. The SPEIbase v2.0 database is based on the FAO-56 Penman–Monteith equation [21,22,23]. Negative SPEI values represent a rainfall deficit below average precipitation and high potential evapotranspiration. Dry episodes begin when the SPEI value is below or equal to −1.0. Positive SPEI values indicate that rainfall surpluses are higher than median rainfall and that potential evapotranspiration is low. The wet periods start when the SPEI value is above or equal to 1.0, and end when the value becomes negative. 

In the last century, the dynamic changes in SPEI can be seen in Figure 3. In the first two decades of the twentieth century, only a few times have the mean SPEI been lower than 0, and this period was rather moisture. In the next six decades, from 1920–1980, much more often the SPEI has been less than 0, so dry periods seem to occur more frequently. Since the 1980s, more frequent and longer dry period, including extremely and exceptionally dry, has been observed. (Figure 2 and Figure 3). 

### 2.1. δ^13^ C and iWUE and Climate Changes

**Figure 4:**
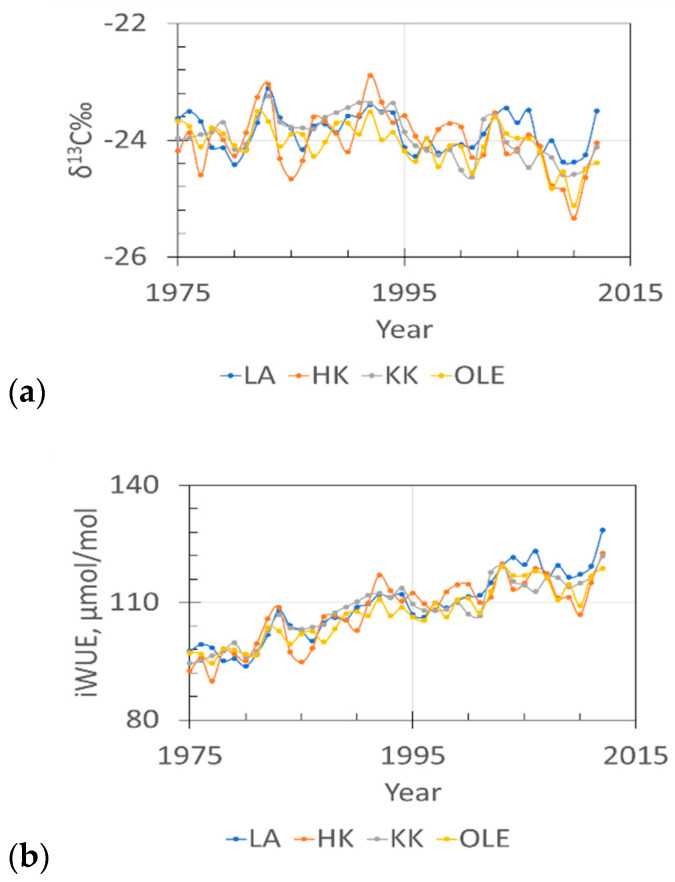
Changes in carbon stable isotope composition of tree rings (**a**) and iWUE (**b**) in pine grown in four forests in Silesia (Poland): HK-Dabrowa Górnicza, LA-Łaziska Górne, KK-Kędzierzyn Koźle, OLE-Olesno.

Figure 4 shows a clear trend of decreasing the value of δ^13^C value, Figure 4b shows an increase in iWUE. 

**Figure 5:**
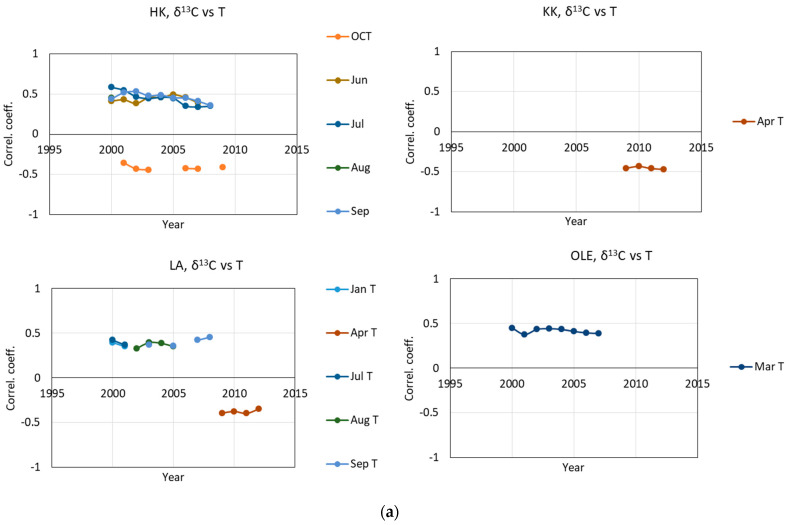

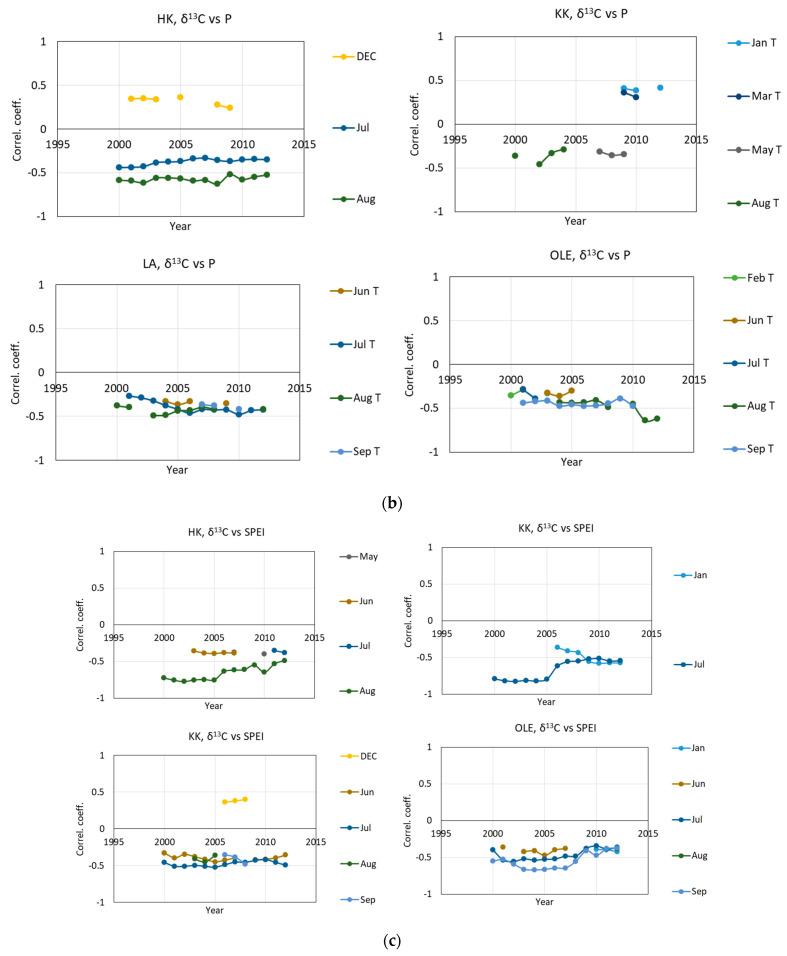

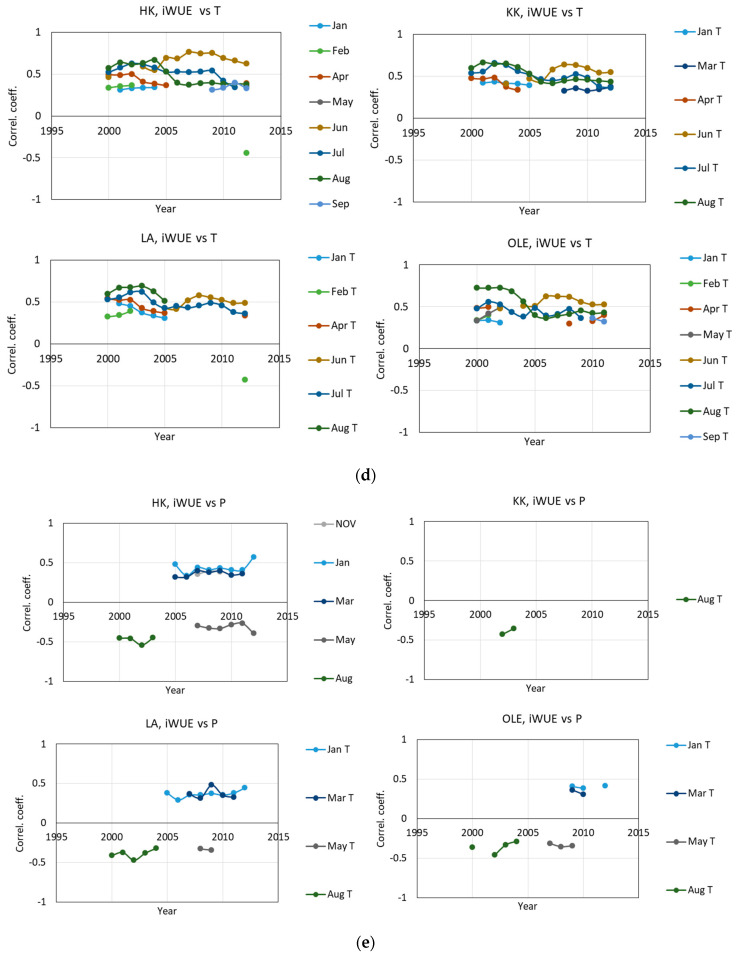

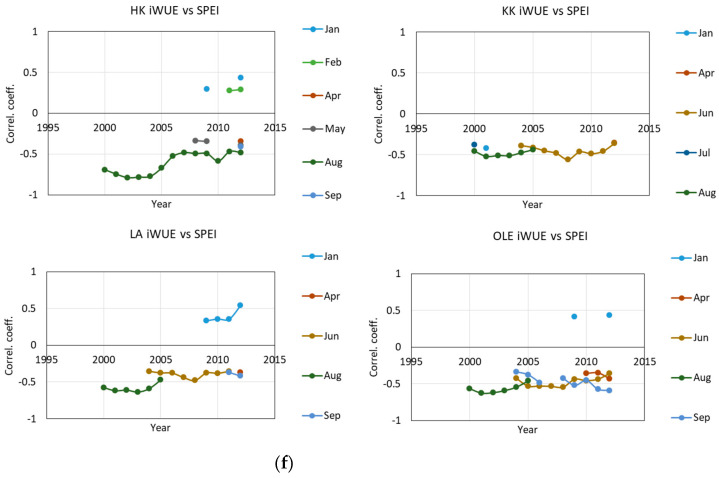
The impact of climate changes on fractionation of carbon isotopes in pine grown in four forests in Silesia (Poland): HK-Dabrowa Górnicza, LA-Łaziska Górne, KK-Kędzierzyn Koźle, OLE-Olesno. Relationship between δ^13^C and monthly mean air temperature (T) (**a**), monthly sum of precipitation (P) (**b**), standardized precipitation−evapotranspiration index (SPEI) (**c**) and iWUE between monthly mean air temperature (**d**), monthly sum of precipitation (**e**) and standardized precipitation-evapotranspiration index (**f**). The results of the temporal stability test of the correlation between climatic parameters and δ^13^C and iWUE based on the bootstrap correlation and the moving interval correlation (base length 26 years, last year was indicated on the *x*−axis). The investigated period of time was from September (capital letter) of the previous year until September of a given year, *p* < 0.05, investigated cover period of time: 1975–2012).

The tests revealed weak and significant correlations between T, P, SPEI, and δ^13^C, iWUE. The results of the moving correlation tests presented in Figure 5 are useful in analyzing the temporal stability of the significant relationships between the selected parameters. The results confirm that there is a high correlation between climatic factors and carbon isotopic composition and iWUE and a high positive or negative relationship between them can be observed over the investigated period of time, whereas the other may be visible only for a short period of time, while the others might be hazardous. At this time, we cannot exclude that some results of the response of trees to climate change may be masked by the anthropopression in this region. 

Figure 5a,b shows the coefficient correlation between δ^13^C and temperature and precipitation, respectively. Figure 5c reveals a relationship between δ^13^C and SPEI. Figure 5d,e shows a strong correlation between iWUE and temperature and precipitation, respectively. Figure 5f reveals a relationship between iWUE and SPEI.

In general, a negative relationship of δ^13^C with precipitation in July and September has been observed, whereas some positive relationship has been observed with the amount of precipitation in winter. However, a negative relationship of δ^13^C with temperature in March and a April and positive relationship with temperature in September has been observed. Comparing δ^13^C with SPEI, a strong significant correlation with SPEI in July has been observed. Most trees show a strong correlation with different summer months (June, July, August, and September). The lower value of SPEI corresponds to an increase in δ^13^C in trees.

The analysis of iWUE gives the possibility to investigate the impact of climate factors on carbon isotopic composition by removing the effect of depletion of ^13^CO_2_ in the air caused by fossil fuel combustion.

The temporal stability tests of the correlation between iWUE and air temperature, amount of precipitation, and SPEI results show a significant relationship, in most of the investigated sites, with temperature in June, July, and August. Interestingly, there are also differences in sensitivity. The strength of this relationship has not been constant and has been modified over an investigated period of time. The correlation between iWUE and temperature is surprising in the last years—where the relationship with spring seems to start significantly. The relationship between winter temperatures (February) is probably a risk. However, this significant relationship between iWUE and temperature in February was noted in two sites. The results of moving correlation tests suggest that iWUE may be affected by the amount of precipitation in winter and spring (January and March), where a positive correlation has been observed, and with precipitation in August (negative relationship). In the last period of time also, the sum of precipitation in May seems to be significant.

The results of moving correlation tests presented in Figure 5 show the strong positive relationship between iWUE and SPEI in summer, in June, August, and September.

### 2.2. Long- and Short-Term Basal Area Increment

**Figure 6:**
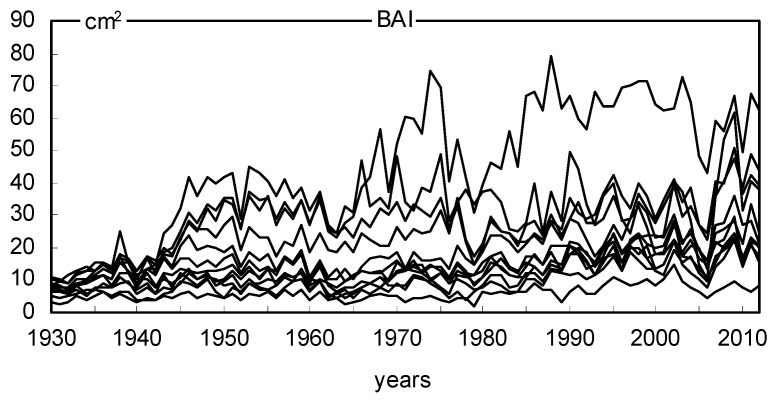
Eighteen site chronologies of basal area increment.

**Figure 7:**
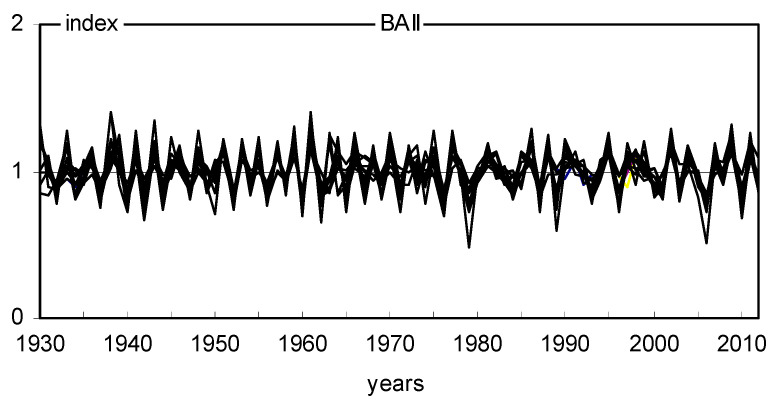
Eighteen site indexed chronologies.

**Figure 8:**
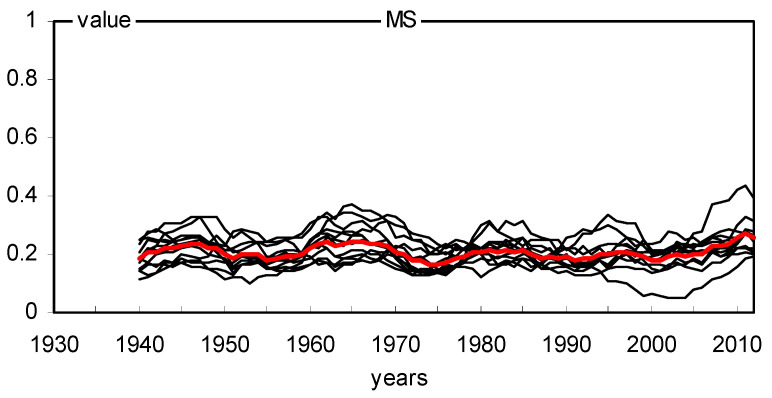
Ten-year running values of MS index. The consecutive MS values are plotted at the end of each 10-year period; the first period ranged from 1931 to 1940 and the last period from 2003 to 2012. The average MS values are marked with a red line.

**Figure 9:**
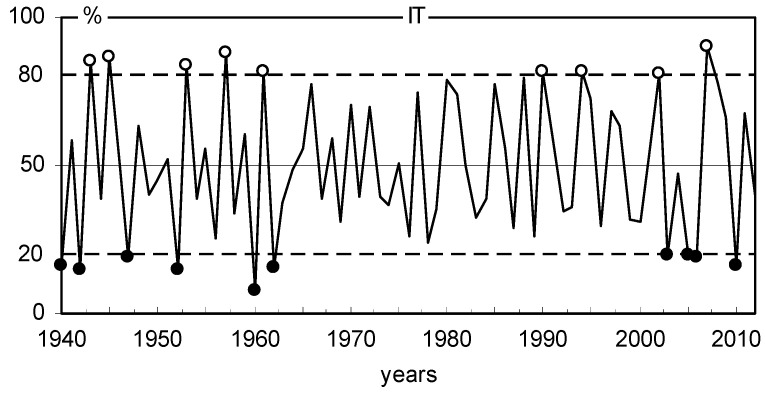
Regional chronology of IT indices and positive (white dots) and negative (black dots) pointer years.

The site chronologies of basal area increments (BAI) are characterized not only by clear short-term variability but also by long-term variation (Figure 6). The chronologies had a similar long-term pattern. The BAI gradually increased with the age of the trees and peaked mostly after several decades. We see long-term BAI growth at all sites. The similarity of annual changes in BAI in 18 pine populations was also relatively high (Figure 6). The variation in BAI from year to year is mainly the result of the effects of climate conditions on trees. Therefore, it can be assumed that the climate signal contained in the chronologies of 18 pine populations was similar during the period studied. The indexed chronologies (BAII) show a strongly reduced long-term variation, while short-term variation is highlighted (Figure 7). MS values indicate high annual sensitivity of trees and strong climatic signals contained in the site BAI chronologies during the period considered. The mean sensitivity (MS) of 18 BAI chronologies in the period 1930–2012 ranged from 0.175 to 0.239, which can be considered relatively high values. The sensitivity of the pines remained relatively high even during periods of high industrial pollution, i.e., the 1960s, 1970s, and 1980s (Figure 8). However, it should be emphasized that pines slightly reduced basal area increments in the 1970s and 1980s (Figure 6), and the heterogeneity of annual increment responses increased. This is evident from the lack of pointer years in the 1970s and 1980s (Figure 9). Before and after this period, homogeneity was much higher. This was probably a consequence of the strong increase in industrial pollution.

### 2.3. BAI, Climate Conditions, and Pointer Years

**Figure 10:**
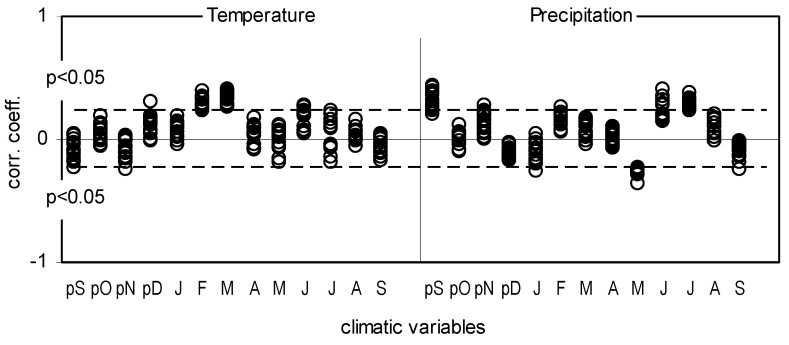
Correlation coefficients between 18 site indexed chronologies (BAII) and mean monthly temperature and precipitation, from September of the previous year (pS) to September of the current year (S).

**Figure 11:**
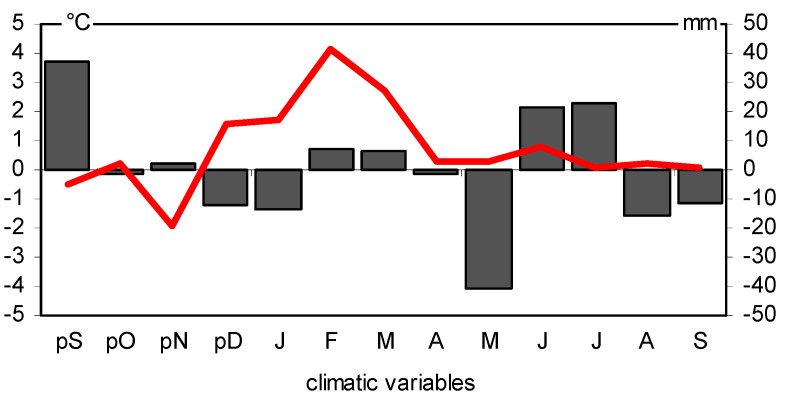
The climatic diagram. Differences in mean monthly temperature (red line) and precipitation (black bars) between the negative and positive pointer years.

A total of 18 site-indexed chronologies (BAII) were correlated with the climate parameters (Figure 10). The relationship between BAI and air temperature in February and March, as well as precipitation in the previous September, and current May and July, was very similar in all pine populations (Figure 10). These correlations are confirmed by the analysis of the climatic conditions in the positive and negative pointer years (Figure 11). The positive pointer years were characterized by high precipitation in September of the previous year, a warm and short winter, low precipitation in May, and high precipitation in June and July in the year of tree-ring formation. In the negative pointer years, the climatic conditions were opposite (Figure 11).

## 3. Discussion

### 3.1. Climatic Factors That Influence the Basal Area Increment

The study area can be considered homogeneous in terms of annual BAI changes in pine trees. Interestingly, the pine populations show a similar sensitivity to climatic conditions even in period of high pollution. However, the heterogeneity of their annual incremental responses increased in this period. This is evidenced by the lack of pointer years. In recent years, there has been an increase in MS, and pointer years have reappeared. This may indicate the increasing sensitivity of pines to climatic conditions. The climatic conditions of the current and the previous year influence tree growth in temperate and boreal zones [27]). This is also the case with Scots pine, as shown by the results of dendroclimatic studies [34,35,36,37,38]. An important factor that affects the increase in the basal area increment of studied Scots pine was the precipitation conditions in September in the year before the current growing season. The high precipitation in this month had a positive effect on wood growth in the following year. Scot pines usually finish their wood growth at the end of August or at the beginning of September [39]. The trees concentrate on accumulating reserve substances that affect the vitality of the trees during the winter period [27]. In turn, sunny weather in September affects the high number of generative organ buds [40,41], and flowering and a lot of cones with seeds in the next spring have a negative impact on wood growth [42]. On the other hand, the cloudy and wet weather in September influences the amount of vegetative buds, and therefore the number of new shoots and needles in the next spring is high [42]. Therefore, the assimilation area and the production of auxins increase, accelerating the cambial division and increasing wood increment. Our results confirm that high precipitation in September has a positive effect on the basal area increment of pines in the following year. 

The results of our study show that warm and short winters also positively influence the condition and wood increment of 18 Scots pine populations. In the temperate zone, bud break and division of the vascular cambium occur after winter. These processes are temperature-dependent [43,44]. The results of many studies show that the earlier onset of wood cell formation in Scots pine trees is related to a higher air temperature at the beginning of the growing season [45,46,47,48]. Interestingly, the dry weather in May had a similar positive effect on the basal area of the pines. These correlations are confirmed in other dendroclimatic studies in pines [35,38,49]. Low precipitation due to lack of cloud cover increases the inflow of direct solar radiation, which warms shoots and needles. The positive effects on the increase in the basal area increment of the pines were also caused by high precipitation in summer, mainly in July in the year of tree-ring formation. It causes a prolongation of the period of intensive divisions of the vascular cambium and the formation of large-diameter cells. The water deficit in summer often leads to earlier termination of cambium activity in conifers [48,49,50]. Therefore, summer droughts are the factor that limits wood growth, which has been observed in the pines throughout their range [51,52,53,54,55,56,57,58].

### 3.2. The Impact of Climatic Changes on δ^13^C, iWUE

The decrease in δ^13^C in trees and the increase in iWUE since 1975–2012 in each sampling site can be explained by isotopic fractionation in the air or the response of the plant to changes in plant physiology due to changes in the ecosystem, for example, changes in weather conditions or water stress and limitation of access to the water. It could be due to climatic changes and also due to the fractionation of δ^13^C in atmospheric CO_2_, which originates from different sources. This phenomenon has been reported by [11,12,13]. It is evident that the emission of CO_2_ from fossil fuel combustion is depleted by ^13^CO_2_. In consequence, mixing natural and anthropogenic CO_2_ in the atmosphere modified the level of ^13^CO_2_ in the atmosphere and a global trend of decrease δ^13^C in the air was observed. Coal was the main fuel in the region, accounting for 83% of the total CO_2_ emissions from fossil fuels in 1950. Recently, gas fuels have increased significantly, and since the late 1990s, gas fuel emissions have exceeded coal emissions. Increased CO_2_ significantly reduces the δ^13^C in the atmosphere. The current δ^13^C is about −8‰.

Other investigations [1,2,3,9,10,11,12,13,15,16,17,18,19,20] conclude from their analyzes that also different climatic factors, tree physiology, and carbon isotope fractionation that occurs due to diffusion in air and carboxylation-causing stomatal conductance, and fractionation (discrimination by RuBisCO) have an influence on carbon isotopic composition of plants. Furthermore, previous isotopic research conducted in this research area focused primarily on single climate factors that influence the isotopic composition of selected saccharides in three of the four forests investigated in the current studies. 

It can be observed that the variation in δ^13^C and iWUE can be associated with different climatic factors such as precipitation or temperature and can be correlated with SPEI. In most cases, the impact of the weather conditions of the previous year has not been observed.

The findings in current studies confirm those of earlier studies, such as the climatic factor having an impact on the carbon isotopic composition of tree rings. The results indicate that, in general, δ^13^C has been positively correlated with the temperature in summer and negatively correlated with the temperature in summer, and negatively correlated with the amount of precipitation in winter and spring and positively with the precipitation in summer. iWUE has been positively correlated with temperature; the highest correlation was observed with temperature in June, July, and August. When comparing iWUE with the monthly amount of rain, it can be observed that prior to 2003, there was a strong correlation with precipitation in August (negative correlation), and a positive correlation between iWUE and the amount of precipitation in January and March seems to be more significant in the last decades.

Dynamic changes in photosynthesis rate and stomata conductance may be affected by different environmental factors. On the one hand, taking into account pollution emissions in this area, it cannot be excluded that until the 1990s, photosynthesis could be limited due to pollution and toxic substances in the air that could be toxic to plants; thus, trees absorbed less CO_2_ from the air by leaves during photosynthesis. On the other hand, the decreasing trend in SPEI suggested that in this region the drought episodes seem to be significant and more frequent, so in consequence, trees could react against water transpiration from the leave by modification of stomata conductance. So, more frequent and more extremely dry periods in the last decades might be reflected in dynamic changes in pine stomata conductivity due to water deficit and thus in carbon stable isotope composition and iWUE variations. 

By comparing the results of the variations in moisture over the past century, moderate, very moist, and exceptionally wet months were observed in the last decades. These results can be explained by the fact that the high value of SPEI can usually be associated with short and intensive rain that could cause floods. In effect, water could not be captured by the soil. The decrease in SPEI associated with dry and water deficit periods corresponds to the summer time in Poland. The relationship of δ^13^C and iWUE with the climatic parameters and SPEI is certainly due to the fact that throughout the year, in Poland, in summer, the most intensive but also very short raining episodes are in summer, and the soil cannot save and retain the water or the water evaporates very quickly due to the high temperature. The increase in iWUE may be associated with a variation in the photosynthesis rate or a change in stomata conductance. A more detailed analysis of iWUE [9,15,16] taking into account changes in the photosynthesis rate (A) and changes in the conductances of the stomata (g) give a possibility to create the hypothesis of the reaction of the different scenario of plants as a response to environmental changes. These scenarios of dynamic changes in iWUE may be possible with a different probability. The iWUE may increase if:

1. The photosynthesis rate increases significantly and the stomata conductance is at the same level or does not significantly
2. The photosynthesis rate is constant, and the stomata conductance is reduced.
3. The photosynthesis rate decreases and the stomata conductance is reduced, but stomata conductance reduction is a much more significant process than a reduction in photosynthesis speed.

These results need to be interpreted with caution because, at this moment, it is hard to say which scenario is the most likely one. Taking into account that (i) in the air there is a lot of CO_2_, and trees have access to CO_2_, and currently, the pollution emission from industry is much lower than in the 1960–1980s, so current conditions are more favorable for photosynthesis and that (ii) SPEI reach much lower value so the reaction of trees will be associated with reduction of stomata conductance to avoid rapid water evaporation, in our opinion scenario 2 or 3 is the most probable. But to confirm this hypothesis a further more detailed analysis should be conducted.

The effect of drought and water stress on pine production in the southern part of Poland is evident. As a consequence of these strong rains, the soil could not restore water for a longer period of time. As a consequence, it has been possible to observe the water deficit that could be responsible for plant reactions against water stress.

## 4. Materials and Methods

### 4.1. Study Area

**Figure 12:**
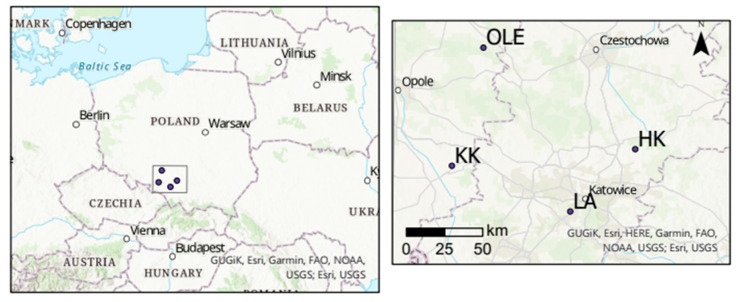
Sampling sites-forests closed to Dąbrowa Górnicza (HK: 50°24′ N 19°28′ E); Kędzierzyn-Koźle (KK: 50°20′ N 18°19′ E), Łaziska (LA: 50°8′ N; 18°53′ E) and a reference site (OLE).

18 pine stands in the following in the forest close to the following cities Dąbrowa Górnicza near steelwork “Huta Katowice” (HK; 50°20′29.7″ N 19°17′04.8″ E), Kędzierzyn-Koźle near chemical factories (KK, 50°18′20″ N 18°15′27″ E), and Łaziska near heat and power station (LA; 50°07′58.0″ N 18°50′47.1″ E) and one reference site (OLE, 50°52′37″ N, 18°25′15″ E) was under dendrochronological investigation (Figure 12). The sampling sites were located at different distances from industrial factories (from 1 to 20 km from the factories). The comparison site was 100 km from industrial factories. On the basis of dendrochronological results, a representative site from each region was isotopically investigated. Selection based on the length and size of the reduction in tree rings width during the most industrialized period of time in Poland [19]. This limitation of the analysis was due to costs and time-limited projects. 

Meteorological data from the meteorological stations in Katowice (50.96° N 19.01° E) and Opole (50.67° N 17.93° E) were provided by the Polish Institute of Meteorology and Water Management (IMGWPIB). The SPEI data was downloaded from the SPEI Global Drought Monitor [21,22,23,24]. 

Using the threshold and symbology for the SPEI https://bennyistanto.github.io/gost-climate/indices/spei.html accessed on 1 October 2022), it is possible to conduct detailed analysis and compare time series, means, extremes, and trends of monthly SPEI with the division to 11 types of period: exceptionally dry for SPEI below or equal to −2.00; extremely dry for SPEI range from −2.00 to −1.50; severely dry for SPEI range from −1.50 to −1.20; moderately dry for SPEI range from −1.20 to −0.70; abnormally dry for SPEI range from −0.70 to −0.50; near normal for SPEI range from −0.50 to +0.50; abnormally moist for SPEI range from +0.50 to +0.70; moderately moist for SPEI range from +0.70 to +1.20; very moist for SPEI range from +1.20 to +1.50; extremely moist for SPEI range from +1.50 to +2.00; exceptionally moist for SPEI equal and above 2.00.

Dendrochronological analyzes cover a period of time from 1900–2012. The relationship between precipitation, temperature, and BAI was analyzed for the period of time between 1951 and 2012, while the relationship between precipitation, temperature, and drought index (SPEI), δ^13^C, and iWUE was analyzed for the period of time between 1975 and 2012. 

### 4.2. Dendrochronological Analysis 

In each of the 18 tree stands (sites), a total of 15 dominant and co-dominant Scots pine trees were selected for this study. The age of trees was about 100 years old. Two increment cores were taken from each tree at 1.3 m above the ground. The cores were sanded and scanned in a resolution of 2400 DPI. The widths of the tree rings in cores were then measured to the nearest 0.01 mm using CooRecorder and CDendro 7.8 software [58]. The quality of the measurement data and the cross-dating was checked with the COFECHA program [59]. For each tree in each year, the mean tree-ring width was calculated as

r = (r_1_ + r_2_)/2 (1)

based on radious r_1_ and r_2_.

The annual basal area increment (BAI) was calculated from the average radius values as follows;

(2) $$BAIi=\pi r_{i}^{2}-\pi r_{i-1}^{2}$$

where: r_i_ is the radius increment of the stem in the year i and r_i–1_ is the radius increment in the previous year.

For each site, the annual BAI of 15 trees sampled in each year was averaged. In this way, the BAI chronologies of the sites were created. In addition, the BAI series of each tree was subjected to indexing and autoregressive modeling using the ARSTAN program [58]. The program twice fits a negative exponential curve or trend line to each BAI series. The BAI indices (BAII) for each year are calculated using the following formula.

BAII_i_ = R_i_/Y_i_ (3)

where R_i_ is the BAI in the year i and Y_i_ is the value of a fitted curve in the year i.

Subsequently, each indexed series was subjected to autoregressive modeling to eliminate autocorrelation. This removed trends, long-term fluctuations, and autocorrelation in the BAI series. Consequently, each tree was represented by an individual BAI and an indexed chronology (BAII). For each pine stand, BAI and BAII chronology was developed based on 15 tree chronologies. The average relative difference in radial increment of the trees was evaluated using mean sensitivity (MS) [27]. The MS values were calculated for the period 1931–2012 and for 10-year periods. The MS determines the sensitivity of trees to short-term environmental stimuli, mainly climate. Correlation coefficients were calculated for each site indexed chronology (BAII) and climate parameters (mean monthly temperature and total monthly precipitation from the previous September to September in the year of tree-ring formation). Furthermore, IT indicators (interval trend indices) were calculated for all (270 = 18 sites × 15 trees) BAI series [60];

IT = 100 · m/n (%)(4)

where: m, the number of trees where BAI in the current year is greater than BAI in the previous year, n, the total number of trees studied.

A higher value of IT corresponds to a higher degree of homogeneity of the incremental response of trees in a given year. IT equal to 100% means that all trees increased BAI compared to the previous year. IT equal to 0% means that all trees decreased BAI compared to the previous year. IT equal to 50% means that half of the trees decreased BAI compared to the previous year and half of the trees increased BAI. The climatic conditions in the years of positive (IT > 80%) and negative (IT < 20%) pointer years were compared to identify the climatic factors that contributed to the occurrence of the pointer years.

### 4.3. Isotopic Analysis

The isotopic chronologies were based on a pooled-ring approach, with 10 trees per each of the four sites (LA, HK, KK, OLE). The sites were chosen during previous research that was immediately related to the current studies [7,8,17,18,19,20]. 

The isotopic composition of the α-cellulose, extracted using the standard procedure [15,18,19,20,21,61] was measured in triplicate samples by IRMS (Isoprime, GV Instruments, Manchester, UK) at the Institute of Physics of the Silesian University of Technology, Poland, and reported with the delta notation in respect to the international standard (VPDB):

δ = (R_sample_/R_standard_ − 1)·1000, ‰. (5)

where R_sample_ and R_standard_ are the molar fractions of ^13^C/^12^C the sample and the standard, respectively. The calibration was performed using an internal standard (C-3 and C-5, IAEA for δ^13^C, standard deviations for each triplicate sample were less than 0.3‰).

To calculate iWUE on the basis of δ^13^C in pine and in air, we used the formula according to [3,61]

(6) $$\Delta^{13}C=\frac{\delta^{13}C_{air}-\delta^{13}C_{pine}}{1+\frac{\delta^{13}C_{pine}}{1000}}$$

(7) $$\Delta^{13}C=a+(b-a)\frac{c_{i}}{c_{a}}$$

(8) $$iWUE=\frac{A}{g}=c_{a}[1-\frac{c_{i}}{c_{a}}]0.625$$

where c_i_ is intercellular CO_2_ concentration, c_a_ is ambient CO_2_ concentration, a = ~4.4‰ is the discrimination against ^13^CO_2_ during CO_2_ diffusion through the stomata, and b = ~27‰ is the discrimination associated with carboxylation.

In this study, a detailed analysis of the sensitivity to variations in air temperature has been carried out, and the sum of precipitation and possible evapotranspiration has been carried out using the bootstrap correlation and the correlation of the moving interval, DendroClim2022 [62] (base length of 26 years, the investigated period of time was September of the previous year until September of the given year, *p* < 0.05, period of time: 1975–2012). Although the variation in SPEI for each of the investigated sites is evident but not very significant, a local value of SPEI was taken into account in a more detailed analysis of the influence of climate on selected pine stands in four forests. 

## 5. Conclusions

The results of this study provide information on the stem growth of Scots pine trees in 18 pine stands growing in polluted areas. The pines studied are sensitive to the various climatic factors that occur in the previous and current year of tree-ring formation. The most important factors that affected the increase in the basal area of Scots pine were pluvial conditions in the previous autumn and current spring, and thermal conditions in the winter of the year in which the annual rings were formed. The increasingly warm winters could have a positive effect on the growth of Scots pine in the future. On the other hand, the factor limiting their wood growth will be the ever-decreasing precipitation in summer. Industrial pollution caused only a small reduction in the wood growth of pines and an increase in the heterogeneity of their growth responses. This fact did not have a strong influence on the relationships between the BAI and climate conditions. 

The main factors influencing carbon isotopic fractionation in pines were thermal and pluvial conditions in different seasons of the year of tree ring formation. This study confirms that the carbon isotope composition provides a useful measure of integrated water use efficiency (iWUE) of pines and, together with BAI can be used to classify the tolerance to the drought of pines growing in the industrial forest area. 

SPEI analysis confirmed that there could be a different scenario of the dynamic tree response to water stress. Father detailed analysis should be conducted to confirm which of two scenarios is more probable: or (1) constant photosynthesis rate and reduction of stomata conductivity (2) decrease in the photosynthesis rate and reduction in the stomata conductivity, in the case where stomata conductance reduction is much stronger than photosynthesis rate limitation.

## Data Availability

Not applicable.
